# Seawater acclimation affects cardiac output and adrenergic control of blood pressure in rainbow trout (*Oncorhynchus mykiss*)—implications for salinity variations now and in the future

**DOI:** 10.1093/conphys/coy061

**Published:** 2018-11-22

**Authors:** Erika Sundell, Daniel Morgenroth, Jeroen Brijs, Andreas Ekström, Albin Gräns, Erik Sandblom

**Affiliations:** 1 Department of Biological and Environmental Sciences, University of Gothenburg, Gothenburg, Sweden; 2 Department of Animal Environment and Health, Swedish University of Agricultural Sciences, Uppsala, Sweden

**Keywords:** α-Adrenergic, arterial blood pressure, vascular resistance, water salinity, euryhalinity, climate change

## Abstract

Greater salinity variations resulting from ongoing climate change requires consideration in conservation management as this may impact on the performance of aquatic organisms. Euryhaline fish exhibit osmoregulatory flexibility and can exploit a wide range of salinities. In seawater (SW), they drink and absorb water in the intestine, which is associated with increased gastrointestinal blood flow. Yet, detailed information on other cardiovascular changes and their control across salinities is scant. Such knowledge is fundamental to understand how fish are affected during migrations between environments with different salinities, as well as by increased future salinity variability. We used rainbow trout (*Oncorhynchus mykiss*) as a euryhaline model species and determined dorsal aortic blood pressure, cardiac output and systemic vascular resistance *in vivo* after chronic freshwater—or SW-acclimation. We also assessed α-adrenergic control of blood pressure using pharmacological tools. Dorsal aortic blood pressure and systemic vascular resistance were reduced, whereas cardiac output increased in SW. α-Adrenergic stimulation with phenylephrine caused similar dose-dependent increases in resistance and pressure across salinities, indicating unaltered α-adrenoceptor sensitivity. α-Adrenergic blockade with prazosin decreased resistance and pressure across salinities, but the absolute reduction in resistance was smaller in SW. Yet, both pressure and resistance after prazosin remained consistently lower in SW. This shows that SW-acclimation lowers systemic resistance through reduced vascular α-adrenergic tone, along with other unknown vasodilating factors. The marked changes in adrenergic regulation of the vasculature across salinities discovered here may have implications for cardiovascular and aerobic performance of fishes, with possible impacts on fitness-related traits like digestion and exercise capacity. Moreover, the evolution of more complex circulatory control systems in teleost fishes compared with elasmobranchs and cyclostomes may have been an important factor in the evolution of euryhalinity, and may provide euryhaline teleosts with competitive advantages in more variable salinity environments of the future.

## Teaser

Climate change may exacerbate salinity variations in aquatic ecosystems, potentially affecting fish performance. We show that long-term seawater exposure in rainbow trout increases cardiac output and reduces blood pressure and vascular resistance through altered α-adrenergic control. Possible impacts of salinity fluctuations on fish aerobic swimming and digestive capacity are discussed.

## Introduction

Global climate change is predicted to affect the salinity and its variability in aquatic environments (Kultz, 2015; Seebacher and Franklin, 2012). For example, exacerbated transient reductions in salinity may be predicted for shallow coastal areas due to increases in precipitation and freshwater (FW) run off (Meier *et al.*, 2012), whereas increases in salinity has been postulated for semi-arid regions due to lower precipitation and increased evaporation (Jeppesen *et al.*, 2015). Such changes in salinity can be challenging and constrain the performance of fish. Euryhaline fishes possess physiological traits that allow them to inhabit a wide range of salinities. Osmoregulatory capacity may, therefore, be of added importance for the ability of fish to cope with future environmental changes (Kultz, 2015), and understanding of the physiological constraints and mechanisms underlying euryhalinity is important to predict future resilience of fish populations and inform management efforts (Cooke *et al.*, 2013; Kultz, 2015; Seebacher and Franklin, 2012).

FW generally has a volume loading effect on fish (i.e. gain of water and loss of ions), whereas seawater (SW) has a volume depleting effect (i.e. loss of water and gain of ions; Olson, 1992; Smith, 1932). These passive effects of water salinity are counteracted by a range of active physiological and behavioural modifications (Marshall and Grosell, 2006). In FW, fish take up ions from the surrounding water via specialized cells in the gills and excrete dilute urine (Evans *et al.*, 2005; Perry *et al.*, 2003; Wood and Bucking, 2010). In contrast, in SW, they actively drink and create an inward directed flow of water through solute-linked water absorption mechanisms in the intestine (Bath and Eddy, 1979; Sundell and Sundh, 2012). Excess ions are then actively excreted across the gills and kidneys (Evans *et al.*, 2005; Grosell *et al.*, 2010), and water is conserved by maintaining low urine volumes (Linhart *et al.*, 1999; Smith, 1930). Collectively, these changes allow euryhaline teleosts to maintain osmotic homeostasis with a constant plasma osmolality of ∼300 mOsm across environmental salinities (Evans, 2008; McCormick and Saunders, 1987; McCormick *et al.*, 1998).

Various cardiovascular adjustments are also important for euryhaline fishes when responding and acclimating to water salinity (Brijs *et al.*, 2015, 2016, 2017). Gastrointestinal blood flow increases at least 2-fold in chronically SW-acclimated rainbow trout due to a combination of increased cardiac output (mediated by increased stroke volume) and an increased proportion of blood flow directed to the gastrointestinal tract (Brijs *et al.*, 2016). This elevation in gastrointestinal blood flow is believed to be essential for the convection of absorbed ions and water, as well as for supplying oxygen and nutrients to metabolically active gastrointestinal tissues (Brijs *et al.*, 2015, 2016). In theory, the elevated gastrointestinal blood flow of trout in SW could be caused either by an elevated arterial blood pressure and/or a reduced gastrointestinal vascular resistance (Olson, 2011). However, the dorsal aortic blood pressure (*P*_DA_) decreased by 11–21% in FW-acclimated trout acutely exposed to SW for 24 hours (Maxime *et al.*, 1991), or short-term acclimated to SW for 2 weeks (Olson and Hoagland, 2008). Nonetheless, knowledge gaps remain as no study has determined the effects of salinity on systemic vascular resistance (*R*_SYS_, the sum of the gastrointestinal and the somatic vascular resistances), and it is largely unknown how the hemodynamic status of fish is affected after more chronic SW exposure. Moreover, the only previous study that recorded blood pressure responses to short-term acclimation to SW used rainbow trout with surgically opened pericardia (Olson and Hoagland, 2008), which is known to negatively affect cardiac performance and blood pressure dynamics (Farrell *et al.*, 1988; Sandblom *et al.*, 2006).

Baseline systemic vascular resistance in teleost fishes is to a great extent determined by the α-adrenergic tone on the resistance vasculature, which is primarily mediated by adrenergic neuronal activity (Sandblom and Axelsson, 2011; Sandblom and Gräns, 2017; Smith, 1978; Smith *et al.*, 1985). Thus, it could be hypothesized that the elevated gastrointestinal blood flow in SW-acclimated trout is mediated by a reduced α-adrenergic vasomotor tone on the gastrointestinal resistance vasculature. Indeed, the gastrointestinal vasculature is under α-adrenergic control because gastrointestinal blood flow is markedly reduced following injection of α-adrenergic agonists (Axelsson and Fritsche, 1991; Axelsson *et al.*, 1989, 2000; Sandblom *et al.*, 2012; Seth, 2010), and changes in gastrointestinal blood flow and gastrointestinal vascular resistance with feeding, exercise and hypoxia are at least partially due to changes in α-adrenergic vasomotor tone (Axelsson and Fritsche, 1991; Seth and Axelsson, 2010; Seth *et al.*, 2008). Interestingly, hypertensive trout fed a high salt diet had a decreased dorsal aortic α-adrenoreceptor mRNA expression along with a blunted *P*_DA_ response to exogenous catecholamines (Chen *et al.*, 2007). While this shows that vascular α-adrenoreceptor density and vascular adrenergic sensitivity can be dynamically regulated in trout, it is unknown how the adrenergic control of blood pressure is affected by acclimation to different water salinities. Thus, there is a need for simultaneous measurements of arterial pressure and flow to resolve how *R*_SYS_ and *P*_DA_ changes with salinity in euryhaline fishes. This information is of importance to understand how cardiovascular and aerobic performance traits of fishes are affected by transient and chronic salinity changes, which may have implications for dispersal and fitness of estuarine and migratory fish species now and in a future with more pronounced salinity variations.

Here, we used rainbow trout (*Oncorhynchus mykiss*, Walbaum 1792) as a euryhaline model species that can tolerate a wide range of salinities while being suitable for *in vivo* cardiovascular recordings. Indeed, some strains of wild rainbow trout (i.e. ‘steelhead’) are anadromous and naturally migrate between FW and SW, whereas most farmed rainbow trout strains can acclimate to SW (Brijs *et al.*, 2017; Quinn and Myers, 2004). Specifically, we measured cardiac output along with *P*_DA_ to calculate *R*_SYS_*in vivo* in chronically FW- and SW-acclimated rainbow trout; with the hypothesis that SW-acclimated trout would exhibit reduced *R*_SYS_ and *P*_DA_. Further, we examined whether differences in *R*_SYS_ and *P*_DA_ across salinities could be explained by altered resistance vessel sensitivity to α-adrenergic stimulation or through changes in intrinsic α-adrenergic tone by using specific α-adrenergic pharmacological tools.

## Methods

### Experimental animals

Rainbow trout (*O. mykiss*) were obtained from a local hatchery (Vänneåns fiskodling, Sweden; see Table 1 for mass and length) and kept in a 1000-l tank with aerated recirculating FW (salinity 0–1 ppt) at 10.5 ± 1.0°C for at least 2 weeks. A subset of 30 fish was subsequently randomly assigned for transfer to another identical 1000-l tank with recirculating aerated SW (salinity 30–33 ppt) at the same temperature (10.5 ± 1.0°C). The fish were then acclimated to their respective salinity treatment for a minimum of 6 weeks prior to experimentation. During the holding and acclimation periods, they were fed three times per week with dry commercial trout pellets (9 mm Protec Trout pellets, Skretting, Stavanger, Norway), but fasted for 1 week prior to surgery and experimentation. Animal handling and surgical procedures were performed in accordance with ethical permit #165-2015, approved by the ethical committee in Gothenburg.

**Table 1:** Morphological characteristics of freshwater- and seawater-acclimated rainbow trout (*Oncorhynchus mykiss*)

	*Freshwater*	*Seawater*
Body mass (g)	331.4 ± 14.0	318.9 ± 14.9
Fork length (cm)	31.3 ± 0.4	31.1 ± 0,4
Condition factor	1.08 ± 0.02	1.06 ± 0.02

Data are presented as means ± SEM (*n* = 11–13). No significant differences were found between acclimation groups for any of the variables.

### Surgery and instrumentation

Individual rainbow trout were anesthetized in FW containing Tricaine methanesulphonate (MS-222, 150 mg l^−1^) buffered with NaHCO_3_ (300 mg l^−1^). Length and weight were determined before placing the fish dorsally on water-soaked foam on a surgical table. The gills of the fish were continuously irrigated with recirculating aerated FW (i.e. for both FW- and SW-acclimated fish) at 10°C containing MS-222 (75 mg l^−1^) buffered with NaHCO_3_ (150 mg l^−1^) throughout the surgery. FW was used as anaesthetic solvent for both acclimation groups since earlier attempts of using SW as solvent for the SW-acclimated group resulted in impaired post-surgical recovery, possibly due to impaired drinking during the anesthetized state. The dorsal aorta was cannulated with a custom-made PE-50 catheter using a steel wire guide (Sandblom and Axelsson, 2006; Smith and Bell, 1964). The cannula was inserted dorsally at a ~45° angle, between the second and third pair of gill arches inside the mouth cavity (Axelsson and Fritsche, 1994). The catheter was filled with heparinized (100 IU ml^−1^) 0.9% saline and exteriorized through the snout and locked in place by a bubble on the catheter (Soivio *et al.*, 1975). The fish was then placed on its side and the operculum and the gill arches were lifted to expose the opercular cavity (Sandblom and Axelsson, 2006). The ventral aorta was gently dissected free without damaging nearby nerves and vessels. A Transonic 2.5PSL flow probe (factory calibrated to 10°C; Transonic systems, Inc, Ithaca, NY, USA) was placed around the aorta with the help of a silk suture (size 4–0). Finally, the probe lead and the catheter were attached to the skin with several silk sutures. After surgery, the fish were immediately placed in the experimental setup, which consisted of individual opaque holding tubes with a volume of ~3 l, floating in a 120-l tank receiving a continuous supply of aerated FW or SW (11 ± 1°C) depending on the acclimation salinity. All fish were allowed a recovery time of at least 40 h before experiments were initiated.

### Experimental protocol

Baseline recordings of cardiac output, heart rate and *P*_DA_ were first performed for a minimum of 2 h at the start of each experiment. When stable baseline conditions had been confirmed, four dosages of the α-adrenergic agonist phenylephrine (10, 30, 60 and 100 μg kg^−1^) and saline (0.9%) as a control, were injected into the dorsal aortic catheter, followed by 0.3–0.4 ml saline (0.9%) to clear the catheter dead space. The administration of phenylephrine dosages was randomized, and an additional 0.3-0.4 ml saline (0.9%) was injected when the peak *P*_DA_ response had leveled off to ensure that all traces from the previous injection was cleared from the catheter. Last, one dosage of the α-adrenergic antagonist prazosin (1 mg kg^−1^) was injected in the same way to obtain a complete α-adrenergic blockade. All injections were administered in volumes of 1 ml kg^−1^ body mass (*M*_b_). While the administration order of phenylephrine and saline injections was randomized for each fish, prazosin was always administered last. Before a new injection was administered, care was taken to allow all cardiovascular variables to return to stable baseline levels. The time for this varied among individuals and injections but was typically never longer than one hour. After the experiments, the fish were killed with a sharp blow to the head.

### Data acquisition and analysis

The dorsal aortic catheter was connected to a pressure transducer (pvb Medizintechnik, Kirchseeon, Germany) that was calibrated against a static water column with the water level in the experimental tank serving as the zero reference. The signal from the pressure transducer was amplified using a 4ChAmp pre-amplifier (Somedic, Hörby, Sweden). The Transonic flow probe was connected to a Transonic flow meter (Transonic systems, Inc, Ithaca, NY, USA). The signals from the flow meter and pressure transducer were relayed to a 16SP PowerLab system (ADInstruments, Castle Hill, Australia) that was connected to a computer with LabChart pro data acquisition software (7.3.2, ADIinstruments). Heart rate was determined from the pulsatile blood flow or pressure recordings using the blood pressure module in LabChart pro. *R*_SYS_ was calculated from *P*_DA_ and cardiac output as:

$$R_{SYS}=\frac{P_{DA}}{cardiac\;output}$$

Stroke volume was calculated as:

$$stroke\;volume=\frac{cardiac\;output}{heart\;rate}$$

Mean values for baseline *P*_DA_, cardiac output and heart rate for each individual were obtained from representative calm periods, which were taken at the end of the initial 2 h baseline recording period. To analyze the responses to α-adrenergic stimulation with the different dosages of phenylephrine, mean values for all cardiovascular variables were taken at the peak blood pressure response after each drug injection. The order of administration for the different dosages of phenylephrine was randomized for each fish. Cardiovascular variables after complete α-adrenergic blockade with prazosin were obtained approximately two hours after the drug had been administered and the blood pressure had reached a new steady state. All mean values were typically calculated as 30 s means.

### Statistical analysis

Statistical analyses were conducted using SPSS Statistics 24 (IBM Corp., Armonk, NY, USA). Independent *t*-tests were used for all comparisons between acclimation groups containing one dependent factor, including all baseline and prazosin treatment analyses, as well as all analyses of the absolute changes induced by prazosin. To assess the general effect of the different dosages of phenylephrine within each acclimation group, as well as the general effect between acclimation groups, a repeated measures ANOVA was used with individuals as subject variables and the dose of phenylephrine as the repeated variable. In the model, we included dose of phenylephrine (0, 10, 30, 60, 100 μg kg^−1^), acclimation group and their interactions as fixed effects. To meet the assumptions of statistical tests, a logarithmic transformation was applied for cardiac output, *R*_SYS_ and *P*_PULSE_, and a square root transformation was applied for *P*_DA_. When the assumption of sphericity in the general linear model analyses was not met, we used Greenhouse–Geiser corrections to interpret if the results were significant. Values are presented as means ± SEM and statistical significance was accepted at *P* ≤ 0.05.

## Results

There were no obvious behavioural differences between acclimation groups as fish from both groups generally remained calm in the experimental setup throughout the recording period. There were no differences in body mass, length or condition factor between acclimation groups (*P* < 0.05; Table 1).

### Effects of salinity on baseline cardiovascular variables

**Figure 1:**
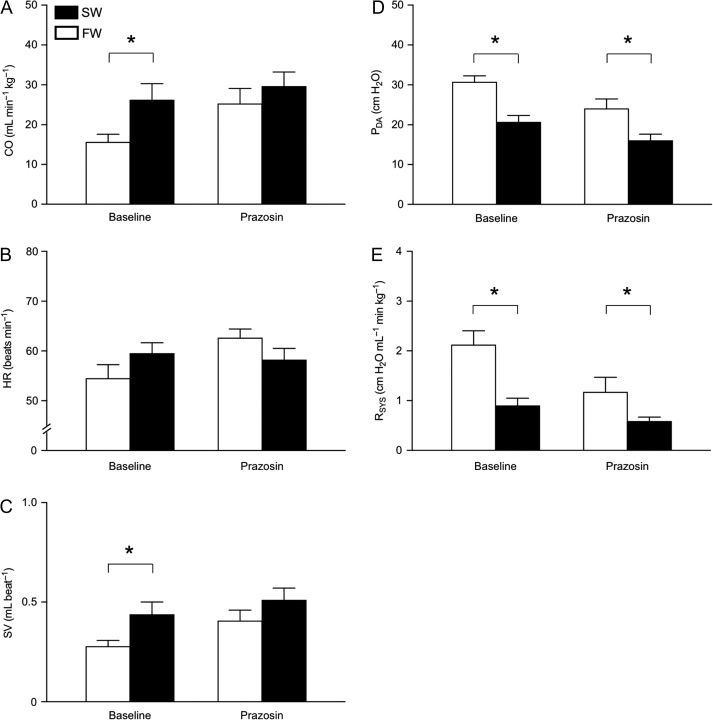
Cardiovascular variables in freshwater- (FW, 0–1 ppt, open bars) and seawater- (SW, 30–33 ppt, closed bars) acclimated rainbow trout (*Oncorhynchus mykiss*). The variables are (**A**) cardiac output (CO, *n* = 8 SW; 9 FW), (**B**) heart rate (HR, *n* = 10 SW; 13 FW), (**C**) stroke volume (SV, *n* = 8 SW; 9 FW), (**D**) dorsal aortic blood pressure (*P*_DA_, *n* = 10 SW; 13 FW) and (**E**) systemic vascular resistance (*R*_SYS_, *n* = 8 SW; 9 FW) during baseline conditions and after α-adrenoreceptor blockade with prazosin (1 mg kg^−1^). Data are presented as means ± SEM. Asterisks (*) denote significant effect of acclimation salinity (*P*≤ 0.05).

**Figure 2:**
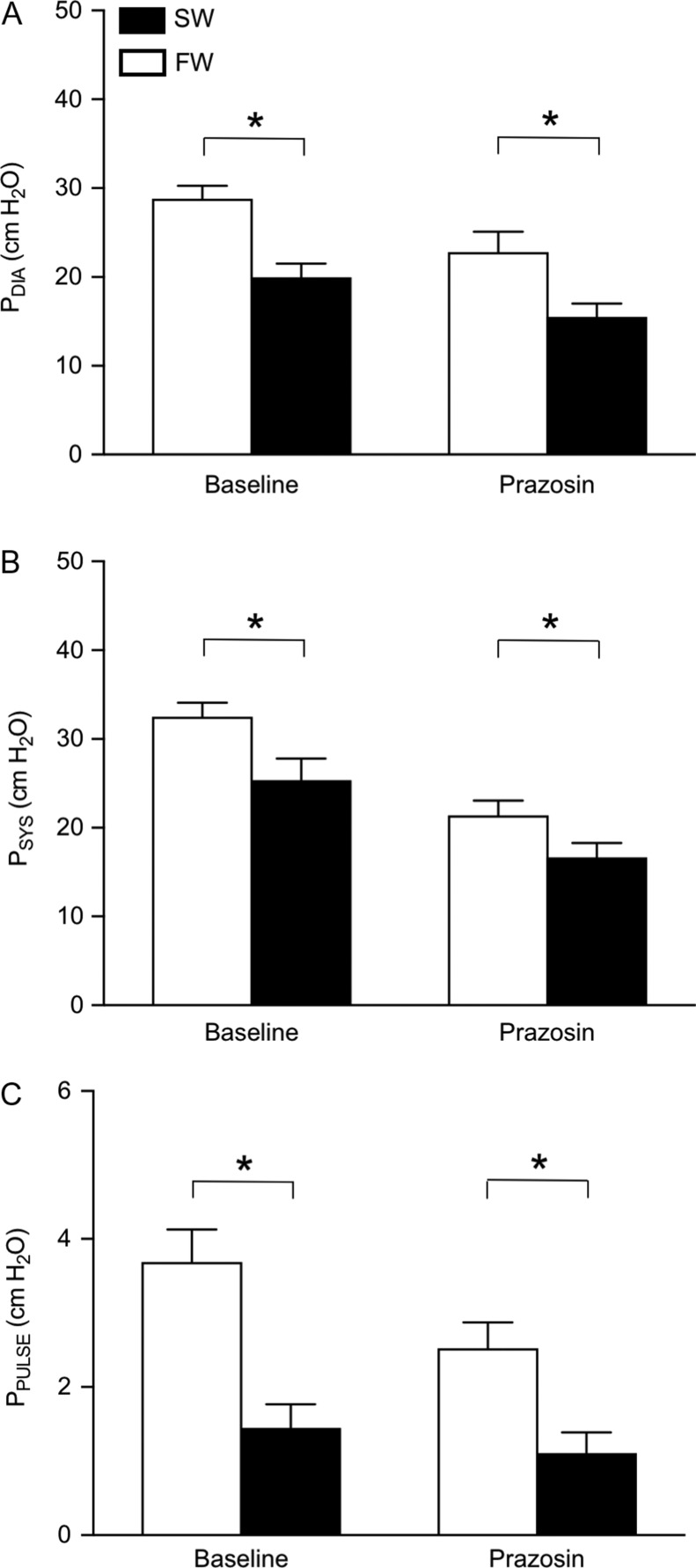
Dorsal aortic blood pressure variables in freshwater- (FW, 0–1 ppt, open bars) and seawater- (SW, 30–33 ppt, closed bars) acclimated rainbow trout (*Oncorhynchus mykiss*). The variables are (**A**) diastolic blood pressure (*P*_DIA_), (**B**) systolic blood pressure (*P*_SYS_) and (**C**) pulse pressure (*P*_PULSE_) during baseline conditions and after α-adrenoreceptor blockade with prazosin (1 mg kg^−1^). Data are presented as means ± SEM (*n* = 10 SW; 13 FW). Asterisks (*) denote significant effect of acclimation salinity (*P* ≤ 0.05).

The SW-acclimated rainbow trout had a significantly higher cardiac output compared to FW-acclimated trout (26.3 ± 4.1 versus 15.7 ± 1.9 ml min^−1^ kg^−1^; *T*_16_ = 2.853, *P* = 0.012; Fig. 1A). The elevated cardiac output in SW was associated with a significantly higher stroke volume (0.44 ± 0.06 versus 0.28 ± 0.03 ml beat^−1^; *T*_16_ = 2.340 *P* = 0.033; Fig. 1C), whereas no significant difference in heart rate between the two acclimation groups was observed (*T*_22_ = 1.423, *P* = 0.169; Fig. 1B). Despite the elevated cardiac output, the SW-acclimated trout exhibited a significantly lower *P*_DA_ (20.7 ± 1.5 cm H_2_O) than FW-acclimated trout (30.8 ± 1.6 cm H_2_O; *T*_22_ = 4.537 *P* < 0.001; Fig. 1D). Consequently, the lower *P*_DA_ of SW-acclimated trout was explained by a significantly reduced *R*_SYS_ (0.90 ± 0.15 versus 2.13 ± 0.28 cm H_2_O min^−1^ ml; *T*_16_ = 4.147, *P* = 0.001; Fig. 1E). The decreased *P*_DA_ in SW was also reflected in significant reductions in dorsal aortic diastolic (*T*_22_ = 4.130 *P* < 0.001; Fig. 2A), systolic (*T*_22_ = 4.820 *P* < 0.001; Fig. 2B) and pulse (*T*_22_ = 3.984 *P* = 0.001; Fig. 2C) pressures.

### Cardiovascular effects of α-adrenergic drugs in FW- and SW-acclimated trout

**Figure 3:**
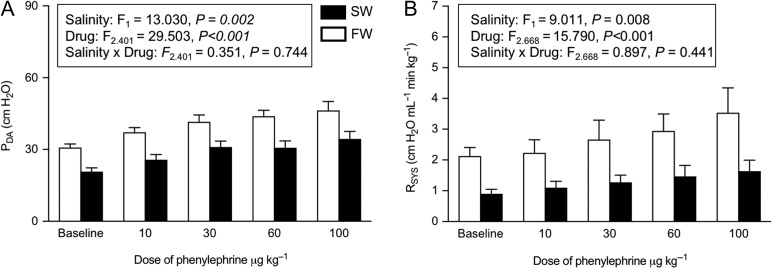
Cardiovascular effects of the α-adrenergic agonist phenylephrine in freshwater- (FW, 0–1 ppt, open bars) and seawater- (SW, 30–33 ppt, closed bars) acclimated rainbow trout (*Oncorhynchus mykiss*). The figures show baseline values and peak responses in (**A**) *P*_DA_ and (**B**) *R*_SYS_ to increasing dosages of phenylephrine (10, 30, 60, 100 μg kg^−1^). Data are presented as means ± SEM (*n* = 11 SW; 13 FW). The inset tables show the statistical outcome from the mixed models for each variable. Statistical significance was accepted at *P*≤ 0.05.

Intra-arterial injection of the α-adrenergic agonist phenylephrine caused dose-dependent increases in *R*_SYS_ and *P*_DA_ in both acclimation groups, but both variables were consistently lower in SW-acclimated trout (Fig. 3A, B). However, when analyzing the absolute changes in *R*_SYS_ and *P*_DA_ from baseline values with each dosage of phenylephrine (data not shown), there were no significant differences between acclimation groups indicating that the responsiveness to α-adrenergic stimulation was unchanged across salinity acclimation groups (*F*_1_ = 2.184, *P* = 0.159 and *F*_1_ = 0.219, *P* = 0.645, respectively).

Phenylephrine injections significantly affected cardiac output (*F*_4_ = 2.974, *P =* 0.026) and stroke volume (*F*_3_ = 16.144, *P <* 0.001) in both acclimation groups (Table 2). Further, administration of phenylephrine typically reduced heart rate in both SW and FW, indicating a barostatic reflex (*F*_2.544_ = 13.059, *P <* 0.001; Table 2). Although not statistically tested, cardiac output appeared to increase at the lower dosages of phenylephrine due to an increased stroke volume, whereas it decreased at higher dosages of phenylephrine as stroke volume reached an upper limit while heart rate continued to decrease (Table 2).

**Table 2:** Cardiovascular effects of α-adrenergic stimulation with phenylephrine in FW- and SW-acclimated rainbow trout (*Oncorhynchus mykiss*)

	Dose of phenylephrine (ml kg^−1^)										
	*Freshwater*					*Seawater*					
Variable	Baseline	10	30	60	100	Baseline	10	30	60	100	Statistics
CO	15.7	19.8	20.7	16.7	16.3	26.3*	27.1	29.1	25.7	25.4	**P:***F*_4_ = 2.974, *P* = 0.026
	±1.9	±2.8	±3.4	±1.9	±1.9	±4.1	±4.0	±5.0	±3.6	±3.8	**S:***F*_1_ = 4.897, *P* = 0.042
HR	54.5	47.7	43.4	43.0	42.5	59.6	54.1	51.9	49.0	44.8	**P:***F*_2.544_ = 13.059, *P* < 0.001
	±2.7	±3.5	±3.6	±3.6	±3.8	±2.1	±3.1	±4.2	±4.0	±4.4	**S:***F*_1_ = 1.716, *P* = 0.204
SV	0.28	0.41	0.42	0.40	0.39	0.44*	0.50	0.56	0.55	0.56	**P:***F*_3_ = 16.144., *P* < 0.001
	±0.03	±0.05	±0.06	±0.04	±0.05	±0.06	±0.06	±0.08	±0.07	±0.08	**S:***F*_1_ = 3.139, *P* = 0.095
*P*_DIA_	28.8	34.0	37.8	39.9	41.7	20.0*	24.4	29.6	28.9	32.0	**P:***F*_2.598_ = 27.274, *P* < 0.001
	±1.5	±1.8	±2.6	±2.2	±3.3	±1.6	±2.0	±2.4	±2.5	±2.9	**S:***F*_1_ = 11.015, *P* = 0.003
*P*_SYS_	32.5	40.5	45.8	48.7	52.3	21.4*	26.9	32.5	32.6	36.9	**P:***F*_2.184_ = 30.316., *P* < 0.001
	±1.6	±2.3	±3.4	±3.0	±4.5	±1.7	±2.3	±2.6	±3.2	±3.4	**S:***F*_1_ = 15.094, *P* = 0.001
*P*_PULSE_	3.69	6.43	8.01	8.81	10.57	1.45*	2.50	2.98	3.73	4.94	**P:***F*_2.530_ = 26.415, *P* < 0.001
	±0.44	±0.74	±1.02	±1.18	±1.66	±0.32	±0.42	±0.46	±0.72	±0.71	**S:***F*_1_ = 25.835, *P* < 0.001

Cardiovascular variables in freshwater- and seawater-acclimated rainbow trout during baseline conditions and after intra-arterial injection with different dosages of phenylephrine (10, 30, 60, 100 μg kg^−1^ body mass). The variables are cardiac output (CO, *n* = 9), heart rate (HR, *n* = 11–13), stroke volume (SV, *n* = 9), dorsal aortic diastolic blood pressure (*P*_DIA_, *n* = 11–13), dorsal aortic systolic blood pressure (*P*_SYS_, *n* = 11–13) and dorsal aortic pulse pressure (*P*_PULSE_, *n* = 11–13). Data are presented as means ± SEM. Asterisk (*) denote significant effects of salinity acclimation on baseline variables. Statistical outcomes for treatment effects of phenylephrine (P) and salinity (S) across phenylephrine concentrations between acclimation groups is denoted in the table. No significant interactions between salinity and phenylephrine treatment were found for any of the cardiovascular variables. Statistical significance was accepted at *P*≤ 0.05.

**Figure 4:**
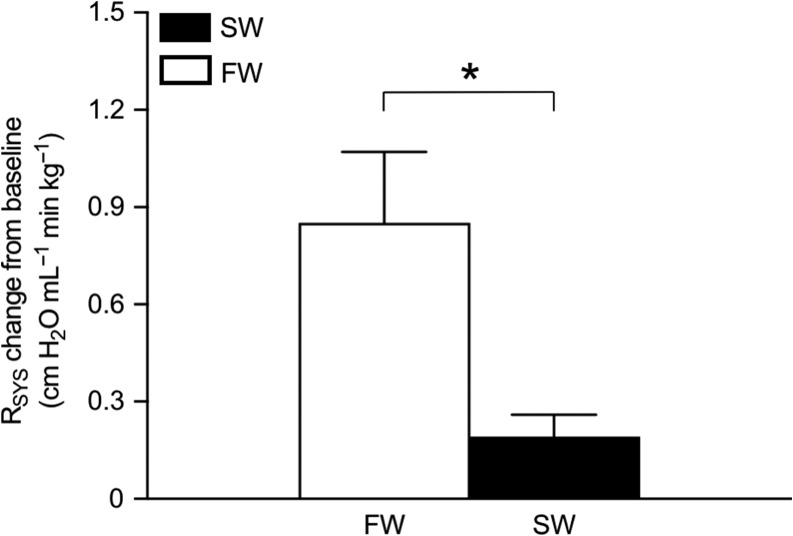
The absolute change in *R*_SYS_ induced by the α-adrenergic antagonist prazosin in freshwater- (FW, 0–1 ppt, open bars) and seawater- (SW, 30–33 ppt, closed bars) acclimated rainbow trout (*Oncorhynchus mykiss*). The figure shows the absolute change from baseline in *R*_SYS_ after prazosin treatment (1 mg kg^−1^). Data are presented as means ± SEM (*n* = 8). Asterisks (*) denote significant difference between salinity acclimation groups (*P*≤ 0.05).

After complete α-adrenoceptor blockade with prazosin, *R*_SYS_ remained consistently lower in SW (*T*_14_ = 2.442 and *P* = 0.028; Fig. 1E), but the absolute reduction in *R*_SYS_ with prazosin was significantly greater in FW- compared to SW-acclimated rainbow trout (*T*_8.312_ = 2.895, *P* = 0.019; Fig. 4). *P*_DA_ was also consistently lower in SW-acclimated trout after prazosin treatment (*T*_20_ = 2.689, *P* = 0.014; Fig. 1D), although the absolute change in *P*_DA_ from baseline with prazosin was not statistically different between acclimation groups. All other blood pressure variables also showed a similar magnitude in the absolute change after prazosin across acclimation groups, but again were consistently lower in the SW-acclimated trout (Fig. 2).

While prazosin induced a significantly greater heart rate increase in FW-acclimated trout compared to SW-acclimated trout (data not shown), there was still no significant difference in heart rate between acclimation groups after prazosin (*T*_20_ = 1.579, *P =* 0.130; Fig. 1B). The magnitude of the absolute changes in stroke volume and cardiac output with prazosin were not significantly different between acclimation groups. However, the clear and significant differences in baseline values for these variables that were observed in untreated trout disappeared with prazosin treatment (cardiac output: *T*_14_ = 0.840, *P =* 0.415 and stroke volume: *T*_14_ = 1.317, *P =* 0.209; Fig. 1A, C).

## Discussion

### Hemodynamic status in FW and SW and possible implications for aerobic performance traits

The present findings demonstrate that *P*_DA_ and *R*_SYS_ are significantly reduced in chronically SW-acclimated rainbow trout. Moreover, our results confirm previous observations of significantly elevated cardiac output in SW-acclimated trout (Brijs *et al.*, 2016, 2017). These fundamental cardiovascular changes in response to salinity open up a range of important questions of how aerobic performance traits such as digestion and swimming capacity are affected by salinity in euryhaline fishes. For example, it is unknown if the elevated cardiac output in SW affects the scope for cardiac output, or whether the cardiovascular system possesses sufficient phenotypic plasticity to compensate across salinities; e.g. by increasing the maximal cardiac output in SW to maintain cardiac scope. While acclimation to different salinities generally has negligible impacts on the maximum swimming capacity of euryhaline fishes (Beamish, 1978; Christensen *et al.*, 2018; Nelson *et al.*, 1996; Wagner *et al.*, 2006), we are not aware of any study comparing maximum cardiac performance during sustained swimming at different acclimation salinities. Thus, while our findings require consideration in conservation management of fish populations that are exposed to varying environmental salinities in their natural habitats, they also highlight the need for further experiments on cardiorespiratory responses to exercise in euryhaline fishes across salinities.

Our data strongly suggest that the elevated gastrointestinal blood flow previously observed in SW-acclimated trout (Brijs *et al.*, 2015, 2016) is caused by a reduced gastrointestinal vascular resistance, since the driving pressure for gastrointestinal blood flow (i.e. *P*_DA_) was markedly reduced in SW and therefore cannot explain the elevated blood flow. However, it is not possible to conclude if a dilation of somatic vascular beds also contributed to the overall reduction in *R*_SYS_, or if somatic vascular resistance increased to aid blood flow distribution to the gastrointestinal tract in SW. Forced feeding of FW and SW-acclimated rainbow trout increased gastrointestinal blood flow with the same absolute amount, which shows that the baseline difference in gastrointestinal blood flow persists after feeding (Brijs *et al.*, 2016). However, whether the elevated gastrointestinal blood flow and decreased α-adrenergic tone on the gastrointestinal resistance vasculature of unfed SW fish constrains the ability to redistribute blood flow away from the gastrointestinal tract to supply swimming muscles during exercise represents another interesting topic to explore in the future.

The reduced *P*_DA_ in SW-acclimated trout was most likely an effect of the reduced *R*_SYS_, which the marked rise in cardiac output was unable to compensate for. However, there are also a few other factors that may have contributed to the decreased P_DA_ in SW. The total circulating blood volume is typically reduced in SW, which could possibly contribute to the reduced blood pressure (Olson, 1992; Olson and Hoagland, 2008). It is also possible that branchial vascular resistance increases with SW-acclimation, which would also reduce the down-stream *P*_DA_. To fully resolve these possibilities, simultaneous measurements of ventral aortic blood pressure and gastrointestinal blood flow in FW- and SW-acclimated trout, along with the cardiac output and P_DA_ measurements performed here, are required.

### Seawater acclimation alters the α-adrenergic control of cardiovascular function

Reduced *R*_SYS_ can result either from elevated vasodilatory and/or reduced vasoconstrictory stimulation of the resistance vasculature (Nilsson, 1994; Olson and Farrell, 2005). The present study examined the α-adrenergic vasomotor tone and found that SW-acclimation of trout leads to reduced α-adrenergic constriction of the systemic resistance vasculature. This decreased α-adrenergic vasoconstriction can either be due to a reduced vascular α-adrenergic sensitivity (e.g. via down-regulation of vascular α-adrenoreceptors), or a reduced intrinsic α-adrenergic neurohumoral tone (Chen *et al.*, 2007; Sandblom and Axelsson, 2011). Both acclimation groups displayed similar dose-dependent increases in *P*_DA_ and *R*_SYS_ with phenylephrine injections, indicating that the vascular sensitivity to α-adrenoreceptor stimulation was similar across acclimation groups. A reduced vascular α-adrenergic sensitivity can therefore not explain the lower baseline R_SYS_ and P_DA_ in the SW-acclimated trout. However, the greater reduction in *R*_SYS_ after α-adrenoreceptor blockade with prazosin in FW-acclimated trout revealed a reduced intrinsic α-adrenergic tone on the resistance vasculature in SW. Consequently, a lower intrinsic α-adrenergic tone at least partly explains the lower baseline *P*_DA_ and *R*_SYS_ in SW. This is likely due to modulation of neural α-adrenergic vascular tone exclusively, as a previous study did not find any significant differences in resting levels of circulating catecholamines between FW- and SW-acclimated trout (Tang and Boutilier, 1988).

Still, the reduced α-adrenergic vasomotor tone cannot alone explain the reduction in *R*_SYS_ in SW, as *P*_DA_ and *R*_SYS_ remained significantly lower in the SW-acclimated trout after complete α-adrenergic blockade. This implies that other local or neurohumoral vasodilatory factors, possibly acting on the gastrointestinal resistance vasculature, are also involved. Likely vasodilating candidates include natriuretic peptides such as atrial natriuretic peptide, which has a strong vasodilating effect on the celiacomesenteric artery in rainbow trout (Cousins and Farrell, 1996; Olson and Meisheri, 1989; Smith *et al.*, 2000), and is released in response to elevated cardiac preload as occurs with SW-acclimation in trout (Brijs *et al.*, 2017; Farrell and Olson, 2000). Indeed, natriuretic peptide secretion increased upon SW transfer in the eel *Anguilla japonica* (Kaiya and Takei, 1996a). This secretion was predominantly triggered by osmotic stimuli from salt loading and cellular dehydration, but also to a lesser extent modified by volume loading (Kaiya and Takei, 1996b, 1997). Other possible vasodilator candidates include nitric oxide and nitric oxide derivatives that act as general vasodilators in the vasculature of teleost fish (Olson and Donald, 2009; Sandblom and Gräns, 2017). In mammals, intestinal hyperosmolarity, as would be expected with SW drinking in fish, have both direct vasodilatory effects on the gastrointestinal resistance vasculature, as well as indirect vasodilatory effects by stimulating nitric oxide production (Bohlen, 1998; Levine *et al.*, 1978; Steenbergen and Bohlen, 1993; Zani and Bohlen, 2005). Interestingly, an increased activity of neuronal nitric oxide synthase was found in the anterior intestine of rainbow trout after 7 days of exposure to SW (25 ppt; Gerber *et al.*, 2018). This indicates a role of nitric oxide in SW-acclimation and as a possible mediator of gastrointestinal vasodilation.

The 68% increase in cardiac output in SW-acclimated trout was primarily mediated *via* an increased stroke volume, as heart rate remained unchanged across salinities (Brijs *et al.*, 2015, 2016, 2017). Nonetheless, the reduction in heart rate induced by phenylephrine in both acclimation groups reveals a functional cardiac baroreflex response at both salinities (Sandblom and Axelsson, 2005). While the increased stroke volume has previously been attributed to a reduced venous capacitance and an increased central venous pressure with SW-acclimation (Brijs *et al.*, 2017), the present study also indicates that the elevated venous pressure may be due to the reduced *R*_SYS_. Interestingly, the significant elevation in baseline cardiac output and stroke volume in SW-acclimated trout was abolished following the prazosin treatment. This could be due to an increased central venous pressure following prazosin treatment in FW-acclimated trout due to altered trans-vascular fluid shifts, as observed in previous *in vivo* studies (see Sandblom and Gräns, 2017).

### Conclusions and perspectives

The present study emphasizes that profound cardiovascular changes occur during acclimation to different salinities in a euryhaline teleost fish. These changes are likely important for maintaining osmotic homeostasis but may impact on aerobic performance traits, which requires future research attention and consideration in conservation management. While the present findings suggest that previous observations of increased gastrointestinal blood flow in SW are due to a reduced α-adrenergic tonus on the gastrointestinal resistance vasculature, our data also indicate that other vasoactive factors are important for mediating these responses. Thus, deciphering the apparently complex interplay between the various neural and hormonal cardiovascular control systems at play during FW to SW transition represents another challenging avenue for further research. From an evolutionary perspective, it could be speculated that the evolution of more complex control systems involving both neural and hormonal vascular control systems in teleost fishes, that are not present in elasmobranchs and cyclostomes (Nilsson, 1983, 1994; Sandblom and Axelsson, 2011; Sandblom and Gräns, 2017), has been an important prerequisite for the evolution of euryhalinity in this diverse group of vertebrates. This evolutionary transition has undoubtedly equipped many teleost species with the physiological machinery necessary to tolerate large acute and chronic salinity changes, as well as the ability to exploit and undertake long-distance migrations across environments with highly contrasting salinities. In fact, this capacity may provide euryhaline teleosts with competitive advantages allowing them to better cope with greater salinity variations in the future resulting from climate change and other anthropogenic perturbations.

## References

[coy061C1] Axelsson M, Fritsche R (1991) Effects of exercise, hypoxia and feeding on the gastrointestinal blood flow in the Atlantic cod *Gadus morhua*. J Exp Biol 158: 181–198. 1717628 10.1242/jeb.158.1.181

[coy061C2] Axelsson M, Fritsche R (1994) Cannulation techniques In Mommsen TP, Hochachka PW, eds Analytical Techniques, vol. 3 Elsevier Science, Amsterdam, pp 17–36.

[coy061C3] Axelsson M, Driedzic WR, Farrell AP, Nilsson S (1989) Regulation of cardiac output and gut blood flow in the searaven, *Hemitripterus americanus*. Fish Physiol Biochem 6: 315–326. 24221601 10.1007/BF01881686

[coy061C4] Axelsson M, Thorarensen H, Nilsson S, Farrell AP (2000) Gastrointestinal blood flow in the red Irish lord, *Hemilepidotus hemilepidotus*: long-term effects of feeding and adrenergic control. J Comp Physiol 170: 145–152. 10791574 10.1007/s003600050269

[coy061C5] Bath RN, Eddy FB (1979) Salt and water balance in Rainbow Trout (*Salmo Gairdneri*) rapidly transferred from fresh water to sea water. J Exp Biol 83: 193–202.

[coy061C6] Beamish FWH (1978) Swimming capacity In Hoar WS, Randall DJ, eds Fish physiology, vol. 7. New York, San Fransisco. Academic Press Ltd, London, pp 101–187.

[coy061C7] Bohlen H (1998) Mechanism of increased vessel wall nitric oxide concentrations during intestinal absorption. Am J Physiol Heart Circ Physiol 44: 542–550. 10.1152/ajpheart.1998.275.2.H542 9683443

[coy061C8] Brijs J, Axelsson M, Gräns A, Pichaud N, Olsson C, Sandblom E (2015) Increased gastrointestinal blood flow: An essential circulatory modification for euryhaline rainbow trout (*Oncorhynchus mykiss*) migrating to sea. Sci Rep 5: 10430 doi:10.1038/srep10430. 26000616 10.1038/srep10430 PMC5377047

[coy061C9] Brijs J, Gräns A, Ekström A, Olsson C, Axelsson M, Sandblom E (2016) Cardiorespiratory upregulation during seawater acclimation in rainbow trout: effects on gastrointestinal perfusion and postprandial responses. Am J Physiol Regul Integr Comp Physiol 310: 858–865. 10.1152/ajpregu.00536.2015 26911464

[coy061C10] Brijs J, Sandblom E, Dekens E, Näslund J, Ekström A, Axelsson M (2017) Cardiac remodeling and increased central venous pressure underlie elevated stroke volume and cardiac output of seawater-acclimated rainbow trout. Am J Physiol Regul Integr Comp Physiol 312: 31–39. 10.1152/ajpregu.00374.2016 27903511

[coy061C11] Chen X, Moon TW, Olson KR, Dombkowski RA, Perry SF (2007) The effects of salt-induced hypertension on α_1_-adrenoreceptor expression and cardiovascular physiology in the rainbow trout (*Oncorhynchus mykiss*). Am J Physiol 293: 1384–1392. 10.1152/ajpregu.00263.2006 17553846

[coy061C12] Christensen EAF, Illing B, Iversen NS, Johansen JL, Domenici P, Steffensen JF (2018) Effects of salinity on swimming performance and oxygen consumption rate of shiner perch *Cymatogaster aggregata*. J Exp Mar Biol Ecol 504: 32–37.

[coy061C13] Cooke SJ, Sack L, Franklin CE, Farrell AP, Beardall J, Wikelski M, Chown SL (2013) What is conservation physiology? Perspectives on an increasingly integrated and essential science. Conserv Physiol 1: 1–23. 10.1093/conphys/cot001 PMC4732437 27293585

[coy061C14] Cousins KL, Farrell AP (1996) Stretch-induced release of atrial natriuretic factor from the heart of rainbow trout (*Oncorhynchus mykiss*). Can J Zool 74: 380–387.

[coy061C15] Evans DH (2008) Teleost fish osmoregulation: what have we learned since August Krogh, Homer Smith, and Ancel Keys. Am J Physiol Regul Integr Comp Physiol 295: 704–713. 10.1152/ajpregu.90337.2008 18525009

[coy061C16] Evans DH, Piermarini PM, Choe KP (2005) The multifunctional fish gill: dominant site of gas exchange, osmoregulation, acid-base regulation, and excretion of nitrogenous waste. Physiol Rev 85: 97–177. 15618479 10.1152/physrev.00050.2003

[coy061C17] Farrell AP, Olson KR (2000) Cardiac natriuretic peptides: a physiological lineage of cardioprotective hormones? Physiol Biochem Zool 73: 1–11. 10685901 10.1086/316727

[coy061C18] Farrell AP, Johansen JA, Graham MS (1988) The role of the pericardium in cardiac performance of the trout (*Salmo gairdneri*). Physiol Zool 61: 213–221.

[coy061C19] Gerber LB, Jensen FBS, Madsen SS (2018) Dynamic changes in nitric oxide synthase expression are involved in seawater acclimation of rainbow trout *Oncorhynchus mykiss*. Am J Physiol Regul Integr Comp Physiol 314: 552–562. 10.1152/ajpregu.00519.2016 29351430

[coy061C20] Grosell M, Farrell AP, Brauner CJ (2010) The role of the gastrointestinal tract in salt and water balance In Grosell M, Farrell AP, Brauner CJ, eds The Multifunctional Gut of Fish. Academic Press, San Diego, pp 135–164.

[coy061C21] Jeppesen E, Brucet S, Naselli-Flores L, Papastergiadou E, Stefanidis K, Nõges T, Nõges P, Attayde JL, Zohary T, Coppens J, et al (2015) Ecological impacts of global warming and water abstraction on lakes and reservoirs due to changes in water level and related changes in salinity. Hydrobiologia 750: 201–227.

[coy061C22] Kaiya H, Takei Y (1996a) Changes in plasma atrial and ventricular natriuretic peptide concentration after transfer of eels from freshwater and seawater or vice versa. Gen Comp Endocrinol 104: 337–345. 8954767 10.1006/gcen.1996.0179

[coy061C23] Kaiya H, Takei Y (1996b) Osmotic and volaemic regulation of atrial and ventricular natriuretic peptide secretion in conscious eels. J Endocrinol 149: 441–447. 8691102 10.1677/joe.0.1490441

[coy061C24] Kaiya H, Takei Y (1997) Interaction of osmotic and volemic mechanisms in secretion of atrial and ventricular natriuretic peptides in eels. Gen Comp Endocrinol 107: 322–326. 9268613 10.1006/gcen.1997.6928

[coy061C25] Kultz D (2015) Physiological mechanisms used by fish to cope with salinity stress. J Exp Biol 218: 1907–1914. 26085667 10.1242/jeb.118695

[coy061C26] Levine SE, Granger DN, Brace RA, Taylor AE (1978) Effect of hyperosmolality on vascular resistance and lymph flow in the cat ileum. Am J Physiol Heart Circ Physiol 3: 14–20. 10.1152/ajpheart.1978.234.1.H14 637909

[coy061C27] Linhart O, Walford J, Sivaloganathan B, Lam TJ (1999) Effects of osmolality and ions on the motility of stripped and testicular sperm of freshwater- and seawater-acclimated tilapia, *Oreochromis mossambicus*. J Fish Biol 55: 1344–1358.

[coy061C28] Marshall WS, Grosell M (2006) Ion transport, osmoregulatioon and acid–base balance In Evans DH, Claiborne JB, eds The physiology of fishes. CRC Press, Boca Raton, pp 177–231.

[coy061C29] Maxime V, Pennec JP, Peyraud C (1991) Effects of direct transfer from freshwater to seawater on respiratory and circulatory variables and acid–base status in rainbow trout. J Comp Physiol B 161: 557–568.

[coy061C30] McCormick SD, Saunders RL (1987) Preparatory physiological adaptations for marine life of salmonids: osmoregulation, growth, and metabolism. Am Fish Soc Symp 1: 211–229.

[coy061C31] McCormick SD, Hansen LP, Quinn TP, Saunders RL (1998) Movement, migration, and smolting of Atlantic salmon (*Salmo salar*). Can J Fish Aquat Sci 55: 77–92.

[coy061C32] Meier M, Andersson HC, Arheimer B, Blenckner T, Chubarenko B, Donnelly C, Eilola K, Gustafsson BG, Hansson A, Havenhand J, et al (2012) Comparing reconstructed past variations and future projections of the Baltic sea ecosystem first results from multi model ensemble simulations. Environ Res Lett 7: 1–8.

[coy061C33] Nelson JA, Tang Y, Boutilier RG (1996) The effects of salinity change on the exercise performance of two Atlantic cod (*Gadus morhua*) populations inhabiting different environments. J Exp Biol 199: 1295–1309. 9319167 10.1242/jeb.199.6.1295

[coy061C34] Nilsson S (1983) Autonomic nerve function in the vertebrates. Springer Verlag, Berlin, Heidelberg, New York.

[coy061C35] Nilsson S (1994) Evidence for adrenergic nervous control of blood pressure in teleost fish. Physiol Zool 67: 1347–1359.

[coy061C36] Olson KR (1992) Blood and extracellular fluid volume regulation: role of the renin angiotensin system, kallikrein-kinin system, and atrial natriuretic peptides In Hoar WS, Randall DJ, Farrell AP, eds Fish physiology, vol. 12B Academic Press, San Diego, New York, London, pp 135–254.

[coy061C37] Olson KR (2011) Circulatory system design: roles and principles In Farrell AP, ed Encyclopedia of Fish Physiology. Academic Press, San Diego, pp 977–983.

[coy061C38] Olson KR, Meisheri KD (1989) Effects of atrial natriuretic factor on isolated arteries and perfused organs of trout. Am J Physiol 256: 10–18. 10.1152/ajpregu.1989.256.1.R10 2521431

[coy061C39] Olson KR, Farrell AP (2005) The cardiovascular system In Evans DH, Claiborne JB, eds The Physiology of Fishes, 3rd edn CRC Press, Boca Raton, pp 119–142.

[coy061C40] Olson KR, Hoagland TM (2008) Effects of freshwater and saltwater adaptation and dietary salt on fluid compartments, blood pressure, and venous capacitance in trout. Am J Physiol Regul Integr Comp Physiol 294: 1061–1067. 10.1152/ajpregu.00698.2007 18184759

[coy061C41] Olson KR, Donald JA (2009) Nervous control of circulation—the role of gasotransmitters, NO, CO, and H_2_S. Acta Histochem 111: 244–256. 19128825 10.1016/j.acthis.2008.11.004

[coy061C42] Perry SF, Shahsvarani A, Georgalis T, Bayaa M, Furimsky M, Thomas SLY (2003) Channels, pumps and exchangers in the gill and kidney of freshwater fishes: their role in ionic and acid-base regulation. J Exp Xool A 300A: 53–62. 10.1002/jez.a.10309 14598386

[coy061C43] Quinn TP, Myers KW (2004) Anadromy and the marine migrations of Pacific salmon and trout: Rounsefell revisited. Rev Fish Bio Fisher 14: 421–442.

[coy061C44] Sandblom E, Axelsson M (2005) Baroreflex mediated control of heart rate and vascular capacitance in trout. J Exp Biol 208: 821–829. 15755880 10.1242/jeb.01470

[coy061C45] Sandblom E, Axelsson M (2006) Adrenergic control of venous capacitance during moderate hypoxia in the rainbow trout (*Oncorhynchus mykiss*): role of neural and circulating catecholamines. Am J Physiol Reg Integr Comp Physiol 291: 711–718. 10.1152/ajpregu.00893.2005 16741138

[coy061C46] Sandblom E, Axelsson M (2011) Autonomic control of circulation in fish: a comparative view. Auton Neurosci 165: 127–139. 21925970 10.1016/j.autneu.2011.08.006

[coy061C47] Sandblom E, Gräns A (2017) Form function and control of the vasculature In Gamperl K, Gillis TE, Farrell AP, Brauner CJ, eds Fish physiology, vol. 36 Academic press, London, pp 369–433.

[coy061C48] Sandblom E, Axelsson M, Farrell AP (2006) Central venous pressure and mean circulatory filling pressure in the dogfish *Squalus acanthias*: adrenergic control and role of the pericardium. Am J Physiol 291: 1465–1473. 10.1152/ajpregu.00282.2006 16825417

[coy061C49] Sandblom E, Davison W, Axelsson M (2012) Cold physiology: postprandial blood flow dynamics and metabolism in the Antarctic fish *Pagothenia borchgrevinki*. PLoS One 7: e33487 doi.org/10.1371/journal.pone.0033487. 22428061 10.1371/journal.pone.0033487 PMC3302773

[coy061C50] Seebacher F, Franklin CE (2012) Determening environmental causes of biological effects: the need for a mechanistic physiological dimenstion in conservation biology. Phil Trans R Soc B 367: 1607–1614. 22566670 10.1098/rstb.2012.0036 PMC3350663

[coy061C51] Seth H (2010) On the regulation of Postprandial Gastrointestinal Blood Flow in Teleost Fish. PhD thesis, University of Gothenburg, Gothenburg, Sweden.

[coy061C52] Seth H, Axelsson M (2010) Sympathetic, parasympathetic and enteric regulation of the gastrointestinal vasculature in rainbow trout (*Oncorhynchus mykiss*) under normal and postprandial conditions. J Exp Biol 213: 3118–3126. 20802112 10.1242/jeb.043612

[coy061C53] Seth H, Sandblom E, Holmgren S, Axelsson M (2008) Effects of gastric distension on the cardiovascular system in rainbow trout (*Oncorhynchus mykiss*). Am J Physiol 294: 1648–1656. 10.1152/ajpregu.00900.2007 18337308

[coy061C54] Smith DG (1978) Neural regulation of blood pressure in rainbow trout (*Salmo gairdneri*). Can J Zool 56: 1678–1683.

[coy061C55] Smith DG, Nilsson S, Wahlqvist I, Eriksson BM (1985) Nervous control of the blood pressure in the Atlantic cod, *Gadus morhua*. J Exp Biol 117: 335–347. 4067502 10.1242/jeb.117.1.335

[coy061C56] Smith HW (1930) The absorption and excretion of water and salts by marine teleosts. Am J Physiol 93: 480–505.

[coy061C57] Smith HW (1932) Water regulation and its evolution in the fishes. Q Rev Biol 7: 1–26.

[coy061C58] Smith LS, Bell GR (1964) A technique for prolonged blood sampling in free-swimming salmon. J Fish Res 21: 711–717.

[coy061C59] Smith MP, Takei Y, Olson KR (2000) Similarity of vasorelaxatant effects of natriuretic peptides in isolated blood vessels of salmonids. Physiol Biochem Zool 73: 494–500. 11009403 10.1086/317732

[coy061C60] Soivio A, Nynolm K, Westman K (1975) A technique for repeated sampling of the blood of individual resting fish. J Exp Biol 63: 207–217. 1159362 10.1242/jeb.63.1.207

[coy061C61] Steenbergen J, Bohlen H (1993) Sodium hyperosmolarity of intestinal lymph causes arteriolar vasodilation in part mediated by EDRF. Am J Physiol 34: 323–328. 10.1152/ajpheart.1993.265.1.H323 8342649

[coy061C62] Sundell K, Sundh H (2012) Intestinal fluid absorption in anadromous salmonids: importance of tight junctions and aquaporins. Front Physiol 3: 1–12. 23060812 10.3389/fphys.2012.00388 PMC3460234

[coy061C63] Tang Y, Boutilier RG (1988) Correlation between catecholamine release and degree of acidotic stress in trout. Am J Physiol Reg Integr Comp Physiol 255: 395–399. 10.1152/ajpregu.1988.255.3.R395 3414834

[coy061C64] Wagner GN, Kuchel LJ, Lotto A, Patterson DA, Shrimpton JM, Hinch SG, Farrell AP (2006) Routine and active metabolic rates of migrating adult wild sockeye salmon (*Oncorhynchus nerka* Walbaum) in seawater and freshwater. Physiol Biochem Zool 79: 100–8. 16380931 10.1086/498186

[coy061C65] Wood CM, Bucking C (2010) The role of feeding in salt and water balance In Grosell M, Farrell AP, Brauner CJ, eds Fish Physiology, vol. 30 Academic Press, London, pp 165–212.

[coy061C66] Zani BG, Bohlen HG (2005) Sodium channels are required during *in vivo* sodium chloride hyperosmolarity to stimulate increase in intestinal endothelial nitric oxide production. Am J Physiol Heart Circ Physiol 288: 89–95. 10.1152/ajpheart.00644.2004 15331363

